# Psychological interventions for post-traumatic stress injuries among public safety personnel: a systematic review and meta-analysis

**DOI:** 10.1186/s13643-022-02112-1

**Published:** 2022-11-25

**Authors:** Anees Bahji, Paula M. Di Nota, Dianne Groll, R. Nicholas Carleton, Gregory S. Anderson

**Affiliations:** 1 Department of Psychiatry, University of Calgary, Calgary, AB Canada; 2 Office of Applied Research and Graduate Studies, Justice Institute of British Columbia, Vancouver, BC Canada; 3 Department of Psychiatry, Queen’s University, Kingston, ON Canada; 4 Department of Psychology, University of Regina, Regina, SK Canada; 5 Faculty of Science, Thompson Rivers University, Kamloops, BC Canada

## Abstract

**Background:**

Public safety personnel (PSP) are exposed to potentially psychologically traumatic events (PPTE) far more often than the general public, which increases the risk for various post-traumatic stress injuries (PTSIs). While there are many evidence-based psychological interventions for PTSI, the effectiveness of each intervention for PSP remains unclear.

**Objectives:**

The current study assessed the effectiveness and acceptability of psychological interventions for PTSI among PSPs.

**Methods:**

A systematic review and random-effects meta-analysis were performed on the effectiveness and acceptability of psychotherapies for PTSIs (i.e., symptoms of depression, anxiety, post-traumatic stress disorder) among PSP. The review adhered to the PRISMA reporting guidelines and used standardized mean differences (Cohen’s *d*), rate ratios (RR), and their 95% confidence intervals (95% CI) to measure pooled effect sizes across studies; negative *d* values and RR values less than one indicated a reduction in symptoms compared to baseline or control groups. In addition, heterogeneity was quantified using *I*^*2*^, and publication bias was evaluated using Egger’s test.

**Results:**

The analyses included data from eight randomized controlled trials representing 402 PSP (79.4% male, 35.3 years). Psychological interventions included narrative exposure therapy (*n* = 1), cognitive behavioral therapy (*n* = 2), eclectic psychotherapy (*n* = 2), eye-movement desensitization and reprocessing (*n* = 1), supportive counseling (*n* = 2), and group critical incident stress debriefing (*n* = 1). The interventions were associated with statistically significant reductions in symptoms associated with PTSD (*d* = − 1.23; 95% CI − 1.81, − 0.65; 7 studies; *I*^*2*^ = 81%), anxiety (− 0.76; 95% CI − 1.28, − 0.24; 3 studies; *I*^*2*^ = 47%), and depression (*d* = − 1.10; 95% CI − 1.62, − 0.58; 5 studies; *I*^*2*^ = 64%). There were smaller but statistically significant improvements at follow-up for symptoms of PTSD (*d =* − 1.29 [− 2.31, − 0.27]), anxiety (*d =* − 0.82 [− 1.20, − 0.44]), and depression (*d =* − 0.46 [− 0.77, − 0.14]). There were no statistically significant differences in dropout rates (RR = 1.00 [0.96, 1.05]), suggesting high acceptability across interventions.

**Conclusions:**

There is preliminary evidence that psychotherapies help treat PTSIs in PSP; however, the shortage of high-quality studies on PSP indicates a need for additional research into treating PTSI among PSP.

**Systematic review registration:**

PROSPERO: CRD42019133534.

## Introduction

Exposure to potentially psychologically traumatic events (PPTE) can lead to many problematic mental health symptoms associated with various disorders, including but not limited to post-traumatic stress disorder (PTSD). Nearly 10% of Canadians meet the criteria for PTSD at any given time [1, 2]. The onset of PTSD in the general population typically occurs in persons in their mid to late twenties [3]. Women appear at twice the risk for PTSD [4, 5]. PTSD can involve substantial distress and impairment [6]. Nearly 75% of patients with PTSD meet the criteria for one or more comorbid psychiatric disorders [7, 8]. PTSD comorbidities exacerbate impairments to quality of life and functioning and are associated with an increased lifetime risk for attempted suicide [4, 7, 9]. PTSD costs the Canadian economy approximately $50 billion annually [10].

Epidemiologic studies have indicated the risk of developing PTSD and other post-traumatic stress injuries (PTSIs) is higher in populations with greater exposure to PPTEs [11–17]. For example, among military personnel and veterans exposed to combat-related violence, the lifetime prevalence of PTSD is as high as 31% [18–23]. Public safety personnel (PSP) are also exposed to PPTE more frequently than the general population [24–30]. The term PSP refers to several related occupations with professionals dedicated to maintaining public safety and wellbeing, such as border services officers, correctional workers, firefighters, paramedics, police, and public safety communicators (e.g., call center operators and 911 operators). The increased PPTE exposures among PSP increase their risk for PTSIs [25, 26, 31–36], including but not limited to PTSD, major depressive disorder, generalized anxiety disorder, and alcohol use disorder [25, 37]. In addition, PSPs are up to four times more likely to experience suicidal behaviors when compared to the general population [35]. The media has increasingly reported mental health risks for PSP, such as responding to the 2014 Moncton shootings [38].

In 2016, the Ministry of Public Safety and Emergency Preparedness at the University of Regina held a national roundtable on PSP mental health. A subsequent pan-Canadian PSP survey estimated that 44.5% of PSPs screened positive for at least one PTSI associated with PPTEs [25, 37]. Additional risk factors for PTSIs among PSP appear to include increased stigma [39, 40], lowered willingness to receive support [33], and difficulties accessing mental health resources [39–41]. The Canadian government has been expanding the scope of tailored mental health programs to serve all PSPs better [42]; however, there are critical knowledge gaps regarding best practices for treating PTSI among PSPs. For example, there is a substantial body of literature on PTSD interventions [43–51], but PSP-specific results remain scarce.

There is very limited research on the effectiveness of psychological interventions for PTSIs among PSPs. Previously published reviews have explored peripheral topics that are of relevance. For example, a recent meta-analysis found a positive but non-significant association between different types of training programs and coping skills among PSP populations [52]. A second meta-analysis found modest evidence for time-limited reductions in PTSI following participation in holistic programs that promote resilience, stress, and emotion regulation among at-risk workers [53]. Another systematic review investigated the effectiveness of organizational peer support and crisis-focused psychological interventions designed to mitigate PTSIs among PSP and other PPTE-exposed workers, producing heterogeneous results and precluding a quantitative meta-analysis [54]. No previous reviews or meta-analyses have examined the effectiveness of psychological interventions for PTSIs among PSPs. Therefore, the current study was designed to conduct a systematic review and meta-analysis of the effectiveness and acceptability of psychotherapies for PTSIs among PSPs.

## Methods

### Protocol and registration

The current study was registered with PROSPERO (CRD42019133534) [55] and reported per the Preferred Reporting Items for Systematic Reviews and Meta-analyses (PRISMA) [56].

### Eligibility criteria

The population-intervention-comparison-outcome-study design (PICOS) model [57] was used to define review eligibility:

- Population: adult (aged 18 and older) police, paramedics and emergency management technicians, correctional officers, dispatchers, 9-1-1 communication officers, and fire and safety officers.
- Intervention: any psychological interventions approved by the American Psychological Association to treat PTSD, delivered alone or in combination with medications.
- Comparison: any comparator condition, such as waitlist controls or no intervention.
- Outcomes: self-report measures (e.g., symptom scores on psychopathology measures), remission for PTSIs (e.g., PTSD), and objective indices of functioning (e.g., absenteeism, occupational performance ratings).
- Study design: English-language, peer-reviewed, randomized controlled trials (RCTs) of any duration from any geographic location published since 2008. We had opted to use 2008 to capture research articles published in the past 10 years at the time of the review’s inception to enrich the review with the most recent literature.

### Information sources and search

A systematic search of Cochrane Central Register, EMBASE, MEDLINE, PsycINFO, and PubMed was performed from January 1, 2008, to October 8, 2019 (Appendix 1). The search was supplemented by reviewing the reference lists of included studies and searching for ongoing RCTs from trial registries.

### Study selection

There were two co-authors (AB and PD) who independently screened all articles by title/abstract and then full texts using Cochrane’s Covidence, a web-based systematic review manager [58]. After removing duplicate citations, the initial screening selection of papers was verified at the title/abstract stage by having multiple reviewers screen 200 reports, with 99% agreement. Finally, all discrepancies were removed by consensus and third-party input (GA).

### Data collection process

There were two co-authors (AB and PD) who independently extracted relevant data from the published full-text reports of each included article using Covidence. The data extracted was verified by one other author (AB, PD, or GA).

### Data items

The following data items were extracted: sample size, age, sex, comorbidity status, and years of employment in the PSP profession; type and duration of psychological intervention; comparator group; all relevant outcome measures (e.g., symptom scores for PTSD and other PTSI on psychopathology measures); author, design, location, and study duration (i.e., timing of follow-up evaluations).

### Risk of bias in individual studies

The Cochrane risk of bias tool for RCTs was used to assess study quality [59]. In brief, the Cochrane risk of bias tool appraises randomization, allocation concealment, blinding, selective reporting, attrition bias, and potential bias from funding. There were two co-authors (AB and PD) who independently appraised each included trial against the risk of bias tool, with discrepancies resolved by consensus.

### Summary measures and synthesis of results

All analyses were performed in RStudio using the *meta* package [60]. Random-effects meta-analysis models were created to pool effect sizes for each psychotherapy's effectiveness and acceptability, and the results were graphed using Forest plots. The specific methods have been previously described [52, 61–65]. The *I*^*2*^ statistic was used to quantify heterogeneity, corresponding to the proportion of total variation (𝜏^2^) not due to random error [66]. Effect sizes were pooled across RCTs using standardized mean differences (SMD, Cohen’s *d*) or rate ratios (RRs) and their 95% confidence intervals (CI), depending on whether the data was continuous or categorical. The SMD was the difference in mean symptom severity scores at the end of intervention between groups divided by the standard deviation of the difference between groups. The SMD allowed the pooling of effect sizes across studies that measured the same construct using different scales, as the standardization corrects for between-scale differences. We harmonized the extraction of means and standard deviations for SMD calculations from the respective studies. A negative SMD indicates an improvement in symptom severity relative to the control condition. All confidence intervals containing zero were non-significant, given that an SMD of zero is null. RRs greater than 1 indicated that the result favored the experimental intervention over the control; 95% CIs containing an RR of 1 were non-significant.

### Risk of bias across studies

Publication bias from the overrepresentation of studies with positive results was assessed using funnel plots [67] and Egger’s funnel plot symmetry test [68, 69].

### Additional analyses

The effect sizes determined from the end-of-intervention were also compared to the end-of-follow-up to determine intervention-time effects.

## Results

### Study selection

**Fig. 1:**
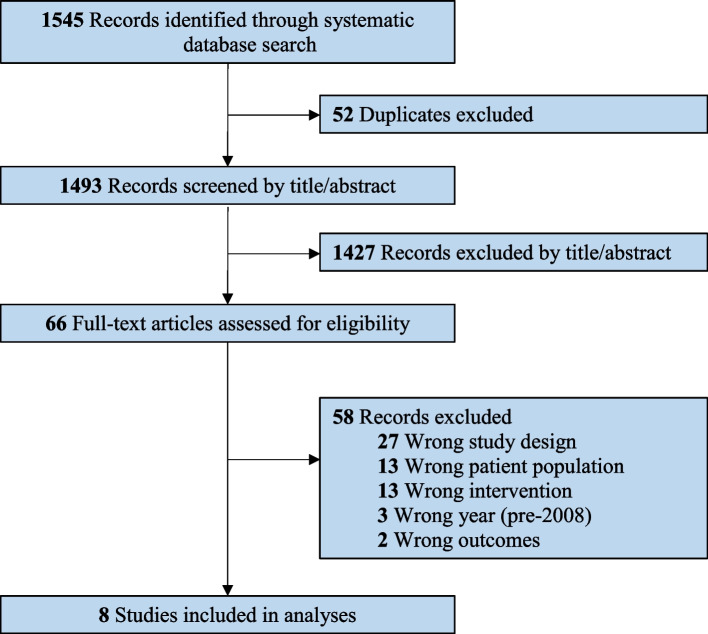
PRISMA flow diagram

The search strategy identified 1493 unique records. After removing 1427 irrelevant documents during the title/abstract phase, the remaining 66 full-text articles were reviewed. The reviewed articles included 27 that involved an ineligible study design, 13 that used an unsuitable intervention, two from before 2008, and 2 that used incompatible outcomes (i.e., they did not report data compatible with a quantitative meta-analysis). Ultimately, eight RCTs met the inclusion criteria for the current review (Fig. 1). We repeated the PubMed search on August 13th, 2022. However, only one additional article was identified, but this study could not be included in the review as it was not a randomized controlled trial.

### Quality assessment

Three RCTs out of the eight [70–72] met the criteria for being high quality as per the Cochrane Risk of Bias tool [70–72]. The remaining five RCTs met low to moderate quality (Table 6 in Appendix 2). Three RCTs out of the eight were double-blinded, while the remaining five were single- or unblinded [70–72]. All but one of the eight RCTs [73] thoroughly reported participant flow and attrition. All eight RCTs adequately randomized participants, but only four concealed intervention allocation [70–72, 74]. All eight studies provided their trial registration numbers and study protocols. Three RCTs out of the eight did not disclose funding sources [73–75].

### Study and participant characteristics

There was considerable diversity in the PSP professions, psychotherapies, and outcome measures across the eight RCTs (Table 1). PSP professions included emergency service personnel, firefighters, and police officers, totalling 402 individuals across studies (79.4% male, mean age 35.3 years). The eight RCTs focused on several different interventions, including narrative exposure therapy (*n* = 1), cognitive behavioral therapy (*n* = 2), eclectic psychotherapy (*n* = 2), eye-movement desensitization and reprocessing (*n* = 1), supportive counseling (*n* = 2), and group critical incident stress debriefing (*n* = 1). Comparator conditions included waitlists (*n* = 3), psychoeducation only (*n* = 2), and non-specific supportive interventions (*n* = 1). Follow-up durations across the eight RCTs ranged from one to 12 months.

**Table 1:** Study characteristics (*n* = 8)

Study	Size	Population	Interventions	Outcomes	Results
Alghamdi et al. 2015 [76]	34	Firefighters	NET (*n* = 17) vs. WLC (*n* = 17), 6 months	SPTSS, retention, HADS	NET reduced PTSD, anxiety, and depression symptoms compared with WLC.
Bryant et al. 2019 [71]	100	First responders	CBT-prolonged (*n* = 33) vs. CBT-brief (*n* = 33) vs. WLC (*n* = 34), 6 months	CAPS, retention, BDI, AUDIT, WHOQOL	CBT is efficacious in reducing PTSD in emergency service personnel.
Chongruksa et al. 2012 [73]	42	Police officers	Group BEP (*n* = 20) vs. psychoeducation (*n* = 22), 1 month	SCL, retention, GHQ-30	Those in the eclectic group counseling had significantly lower symptom scores
Gersons et al. 2013 [72]	42	Police officers	BEP (*n* = 22) vs. WLC (*n* = 20), 3 months	PTSD remission, retention, SCL, AUD remission	BEP improved PTSD symptoms, work resumption, and some comorbid conditions.
Jarero et al. 2013 [75]	39	First responders	EMDR (*n* = 19) vs. supportive counseling (*n* = 20), 3 months	SPRINT, retention	EMDR significantly reduced PTSD scores at the post-test and during the follow-up.
Miller et al. 2019 [77]	71	Police officers	Neuropsychological trauma processing (*n* = 43) vs. single session (*n* = 28), 11 months	PCL-5, retention	No significant reduction in PTSD scores between groups
Mithoefer et al. 2018 [70]	26	Firefighters and police officers	MDMA-assisted psychotherapy: 125 mg (*n* = 12) vs. 75 mg (*n* = 7) vs. 30 mg (*n* = 7), 12 months	CAPS, retention, BDI, GAF	MDMA-assisted psychotherapy reduced PTSD symptoms in a dose-dependent manner.
Tuckey et al. 2014 [74]	48	Firefighters	Group CISD (*n* = 20) vs. education (*n* = 28), 1 month	IES, K10, alcohol use, quality of life	CISD reduced alcohol use and improved quality of life.

*CAPS* Clinician Administered PTSD Scale, *IES* Impact of Events Scale, *K10* Kessler 10, *CISD* Critical Incident Stress Debriefing, *SPTSS* Scale of Post-traumatic Stress Symptoms, *HADS* Hospital Anxiety and Depression Scale, *NET* narrative exposure therapy, *WLC* waitlist control, *CBT* cognitive behavioral therapy, *AUDIT* Alcohol Use Disorders Identification Test, *BDI-II* Beck Depression Inventory 2nd Edition, *WHOQOL* World Health Organization Quality of Life, *SCL-90* Symptom Checklist 90, *GHQ-30* General Health Questionnaire, *AUD* alcohol use disorder, *BEP* Brief Eclectic Psychotherapy, *SPRINT* Short PTSD Rating Interview, *EMDR* Eye Movement Desensitization and Reprocessing, *PCL-5* PTSD Checklist for DSM-5, *GAF* Global Assessment of Function

### Psychotherapy effectiveness for PSP

**Fig. 2:**
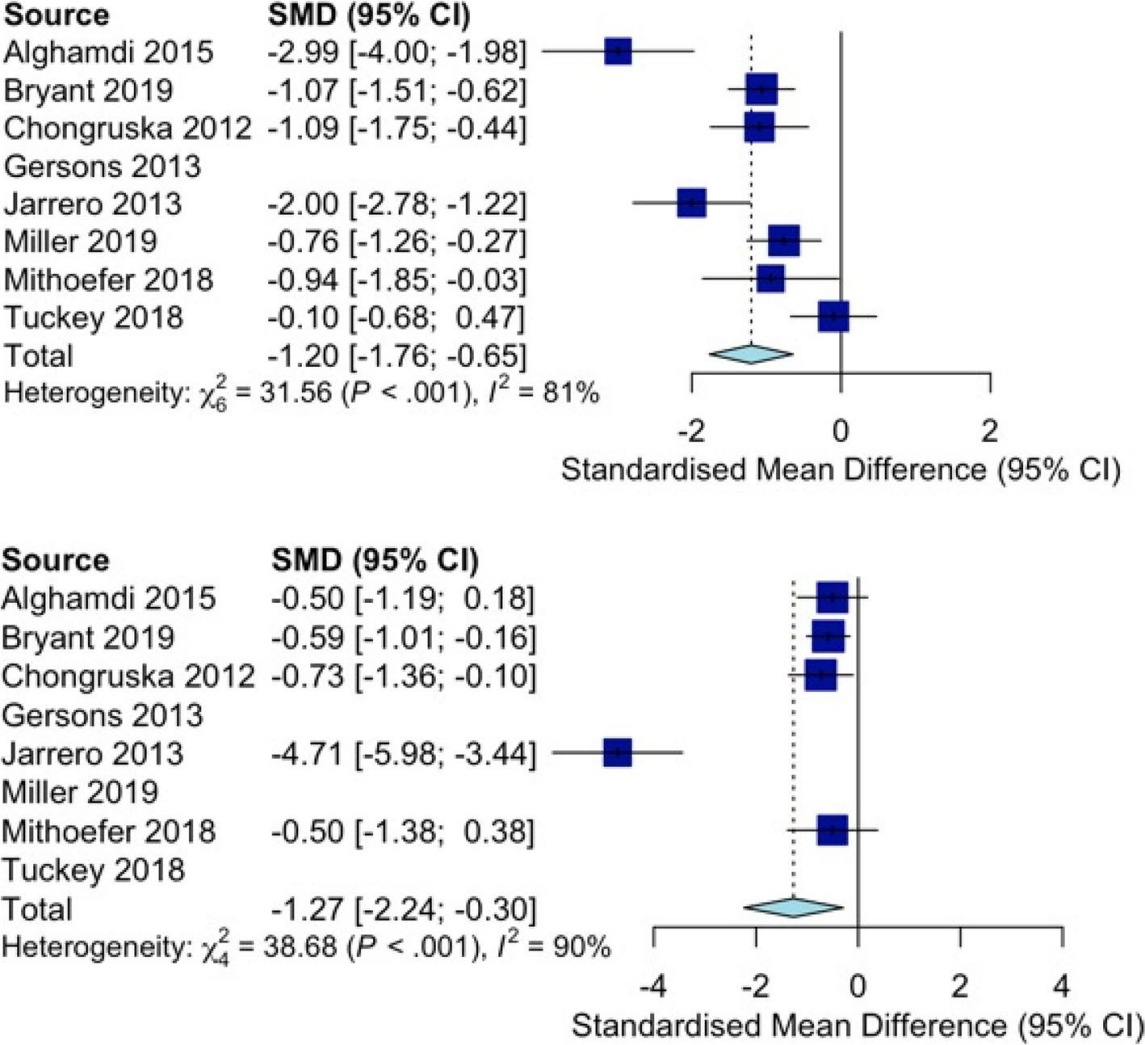
Forest plots for psychotherapies’ effectiveness in reducing PTSD symptom severity after the intervention (top) and follow-up (below)

**Fig. 3:**
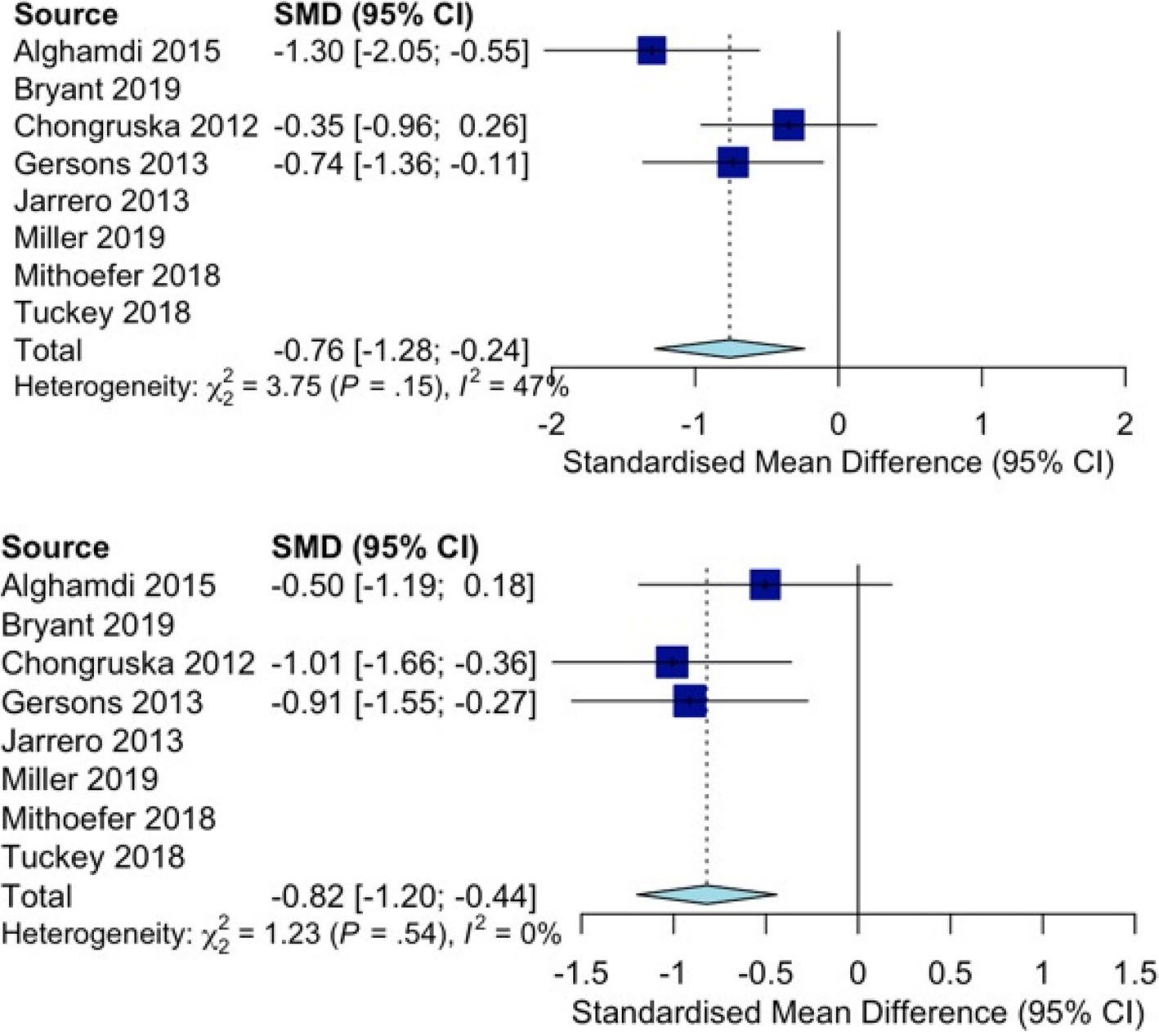
Forest plots for the effectiveness of psychotherapies for reducing anxiety symptom severity after the intervention (top) and in follow-up (below)

**Fig. 4:**
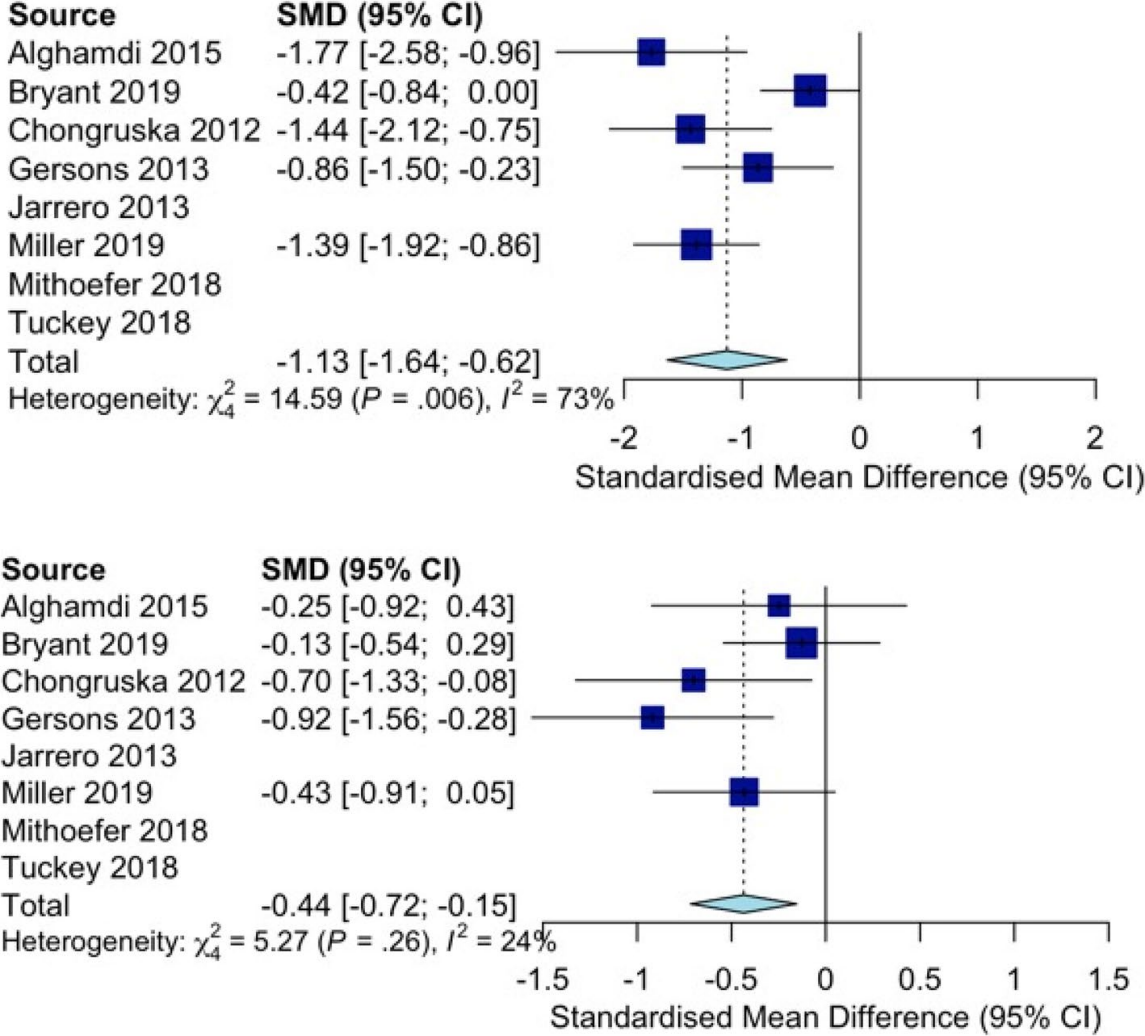
Forest plots for psychotherapies’ effectiveness in reducing depression symptom severity after the intervention (top) and follow-up (bottom)

The included RCTs evidenced reduced PTSD symptom severity at the completion of intervention (*d* = − 1.23; 95% CI − 1.81, − 0.65; 7 studies; *I*^*2*^ = 81%) and in sustained follow-up (*d* = − 1.29, 95% CI − 2.31, − 0.27; 6 studies; *I*^*2*^ = 89%; Fig. 2). The interventions appeared effective for reducing PTSD symptoms at intervention completion (RR = 1.81, 95% CI 1.18–2.79; 2 studies; *I*^*2*^ = 0%) that were sustained at follow-up (RR = 2.15, 95% CI 1.13–4.11; *I*^*2*^ = 29%). There were also statistically significant reductions in anxiety symptoms at intervention completion (*d* = − 0.76; 95% CI − 1.28, − 0.24; 3 studies; *I*^*2*^ = 47%) that were sustained at follow-up (*d* = − 0.82, 95% CI − 1.20, − 0.44; *I*^*2*^ = 0%; Fig. 3). There were also statistically significant reductions in depressive symptoms at intervention completion (*d* = − 1.10; 95% CI − 1.62, − 0.58; 5 studies; *I*^*2*^ = 64%) that were sustained at follow-up (*d* = − 0.46, 95% CI − 0.77, − 0.14; *I*^*2*^ = 15%; Fig. 4). All meta-analytic estimates are summarized in Table 7 in Appendix 3.

### Acceptability of psychotherapies

**Fig. 5:**
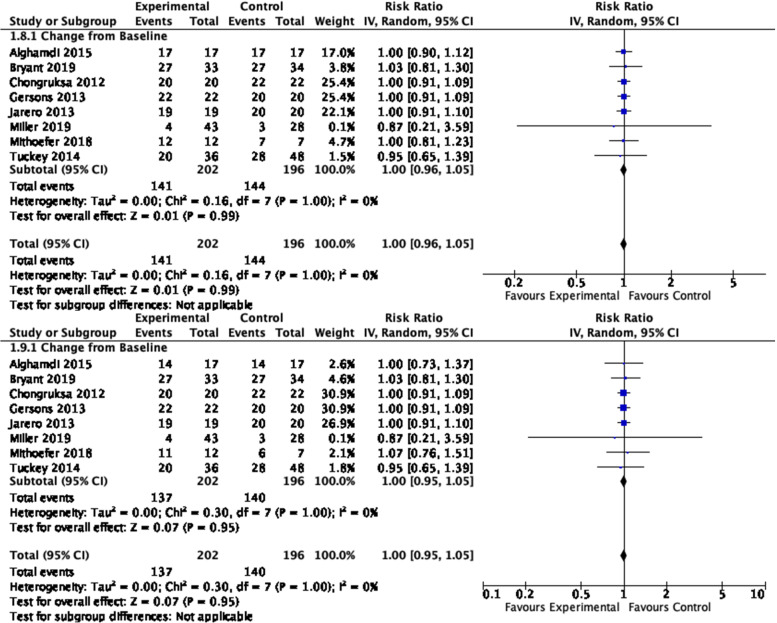
Forest plots of meta-analyses for retention in intervention at (top) primary trial endpoint; (bottom) in the follow-up

There were no statistically significant differences in retention at intervention completion (RR = 1.00; 95% CI 0.96, 1.05; 8 studies; *I*^*2*^ = 0%) or at follow-up (RR = 1.00, 95% CI 0.95, 1.05; *I*^*2*^ = 0%; Figure 5 in Appendix 4).

### Risk of bias across studies

**Fig. 6:**
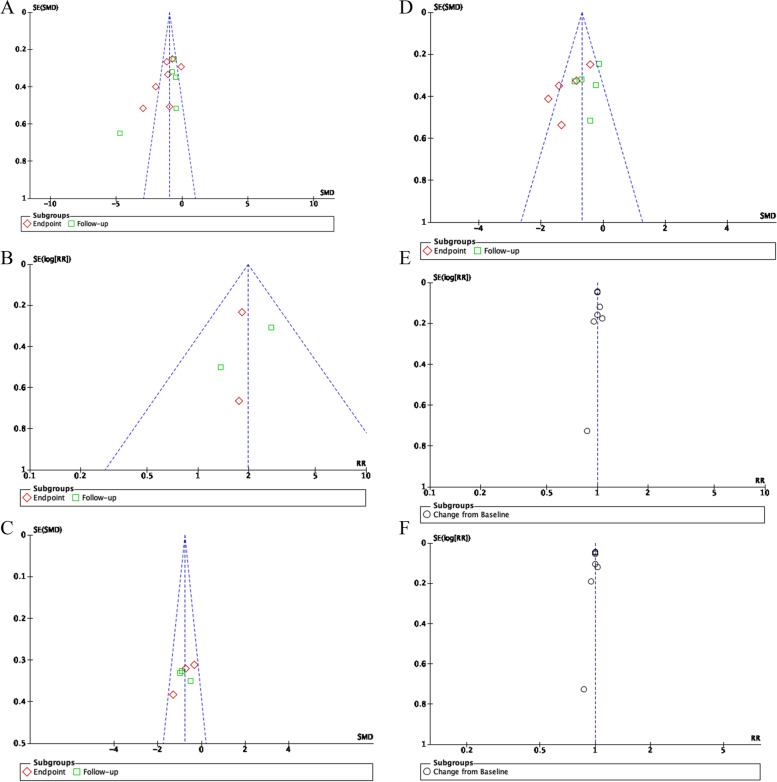
Funnel plots for publication bias in meta-analyses for the effectiveness of psychotherapies for **A** PTSD symptom severity after intervention and follow-up; **B** PTSD remission after the intervention and in follow-up; **C** anxiety symptom severity after intervention and follow-up; **D** depression symptom severity after the intervention and in follow-up; **E** retention in intervention at the primary trial endpoint and **F** retention in intervention at extended follow-up

The overall risk of publication bias appears low because all funnel plots were grossly symmetric, and none of the quantitative tests for publication bias reached statistical significance (Figure 6 in Appendix 5).

### Additional analyses

The low number of RCTs eligible for inclusion (*n* = 8) prohibited conducting subgroup and meta-regression analyses.

## Discussion

The current study is the first meta-analysis of psychotherapeutic interventions for PTSIs among PSPs. The results supported the effectiveness of narrative exposure therapy, cognitive behavioral therapy, eclectic psychotherapy, eye movement desensitization and reprocessing, and trauma processing therapy for PSPs experiencing PTSD, depression, or anxiety symptoms. For most interventions, the effects were sustained at follow-up, indicating the durability of benefits. Across the studies, there was minimal attrition, supporting the interventions’ acceptability among participating PSPs. The evidence suggests several effective and acceptable psychotherapies for PTSD, depression, and anxiety symptoms among PSP. Ultimately, our current results represent an updated synthesis of the literature on the role of psychological interventions for PTSIs among PSP and can serve as a starting point for more high-quality research that could expand on some initial results.

Ongoing advances in our understanding of PTSD and trauma-focused interventions are occurring alongside increasing recognition that professionals with extensive PPTE exposures (e.g., military, veterans, PSP) are experiencing high levels of PTSI. There are also similarities and occupation-specific needs when managing PPTE sequelae among diverse professional groups. For example, two recent reviews found limited evidence supporting the effectiveness of proactive (e.g., resilience promotion) [53] and post-exposure peer support and crisis-focused psychological interventions (e.g., critical incident stress debriefing, stress management, peer support, psychological first aid, trauma risk management) in mitigating PTSIs among PSP and frontline healthcare personnel [54]. The extant literature for PTSI management among PSP includes multiple therapeutic approaches, study designs (i.e., experimental, observational), and outcome measures (e.g., psychopathology self-report measures, clinician-rated functional assessments, and occupational indices such as absenteeism) [54]. Advancements have been made over the past few decades in understanding and treating symptoms of PTSD. Still, the rising number of professionals repeatedly exposed to PPTE continues to be a serious international public health problem, especially in light of operating during the global COVID-19 pandemic [78]. PPTE exposure is nearly ubiquitous [79, 80]. Nevertheless, individuals with more frequent PPTE exposures are at increased risk for PTSIs [81]. For example, approximately 20% of the two million troops deployed to Iraq may have required intervention for PTSD, whereas the population prevalence of PTSD among the deployed troops is less than 10% [82].

Most studies with PPTE-exposed participants have focused on military and veteran populations. There are fewer studies exploring PSP. Accordingly, results from military and veterans are often extrapolated to PSP. There is evidence that first-line PPTE-focused interventions (e.g., cognitive processing therapy, prolonged exposure therapy) produce clinically meaningful improvements for military personnel with PTSD; however, non-response rates among military personnel appear much higher than in civilians [83]. In addition, military participants in PTSD intervention studies who initially respond to intervention report poorer long-term follow-up outcomes than civilian participants [84]. Overall attrition rates appear similar between military and civilian participants receiving intervention for PTSD; however, particular subgroups, such as persons with PTSD related to combat or assault, tend to have poorer intervention outcomes, and are more likely to drop out of follow-up [84]. The relatively lower response to interventions for PTSIs among military and veteran populations, coupled with the near absence of RCT evidence with PSP, underscores the need for additional research and intervention development for people repeatedly exposed to PPTE. The knowledge base regarding PPTE exposures and PTSIs is rapidly expanding with novel research and public priorities to support PSP.

## Limitations

As with any review, the current work has limitations that contextualize the present results’ generalizability and provide future research directions. First, the eligibility criteria limited the included research results to RCTs to minimize bias from confounding variables. High-quality psychotherapy trials are scarce due to inherent methodological challenges such as patient selection, outcome criteria, lack of controls, and difficulty with blinding [85–87]. Only three of the eight RCTs included in the current meta-analysis met the high-quality criteria per the Cochrane Risk of Bias tool [70–72]. Pooling outcomes across potentially heterogeneous populations may have violated meta-analytic assumptions; however, most heterogeneity indices were low, supporting the decision to pool interventions and outcomes across studies. Given the variable trial quality across studies, estimations of intervention effectiveness may have been inflated. Publication bias was low, but restricting the search strategy to English-language articles published after 2008 may have excluded relevant studies. Finally, few studies provided measurements of long-term outcomes, the longest of which was at 12 months of follow-up, limiting assessments of sustained impact. PSPs who receive intervention are very likely to be re-exposed to numerous subsequent PPTE, which suggests the impact of any single intervention may be offset by one or more new PTSI, which necessarily hampers interpretations of sustained intervention impact.

## Future studies

There is a need for ongoing research on PTSIs among PSP [23, 88]. The current review focused on RCTs, but non-RCT designs remain a potentially valuable source of information for advancing the state of knowledge on PTSIs among PSPs. For example, a non-randomized study by Berking and colleagues found evidence that police officers who received manualized emotion regulation training demonstrated superior skill application and improved subject mental health ratings [89]. Additional randomized and non-randomized trials are needed to inform further intervention modalities and delivery models that may be particularly beneficial for PSP, such as internet-based cognitive behavior therapy [31, 42].

## Conclusions

There is preliminary evidence that psychotherapies help treat PTSIs in PSP; however, the shortage of high-quality studies justifies a need for additional studies investigating the intervention of PTSIs among PSP.

## Data Availability

Data will be made available upon request on ResearchGate and Mendeley.

## Appendix 1

### Search strategy

Tables 2, 3, 4, and 5

**Table 2:** PubMed: September 28, 2019

Step	Search	Hits
1	((((((((public safety person*) OR public safety profes*) OR firefight*) OR police*) OR dispatch) OR paramed*) OR first respon*) OR correction*) OR prison*	267,221
2	((((((((((operational stress inj*) OR PTSD) OR posttraumatic*) OR depress*) OR anxi*) OR substance abuse*) OR substance use disorder) OR addiction) OR chronic pain) OR insomnia*) OR sleep disorder*	1,108,096
3	(((((((((((cognitive behavioural therapy) OR CBT) OR cognitive processing therapy*) OR therapy*) OR psychotherapy*) OR eye movement*) OR EMDR) OR present-centred therapy) OR prolonged exposure) OR psychological debriefing) OR stress inoculation training) OR seeking safety	5,092,500
4	1 and 2 and 3	4121
5	Limit 4 to clinical trial	477
6	Limit 5 to the past 10 years	294
7	Limit 6 to humans	292
8	Limit 7 to adults (19+)	253
9	Limit 8 to English	226

**Table 3:** MEDLINE: September 28, 2019

Step	Search	Hits
1	first responder.mp or exp Emergency Responders/	12,272
2	exp Police/	4900
3	exp Firefighters/	912
4	exp Prisons/ or correctional officers.mp.	9890
5	exp Emergency Medical Dispatcher/	35
6	paramedic.mp. or exp Allied Health Personnel/	49,584
7	1 or 2 or 3 or 4 or 5 or 6	65,970
8	exp Depressive Disorder, Major/ or exp Stress Disorders, Post-Traumatic/ or exp Mental Disorders/ or operational stress injury.mp.	1,192,424
9	exp Anxiety Disorders/	77,182
10	exp Mood Disorders/	117,687
11	substance abuse.mp. or exp Substance-Related Disorders/	284,709
12	suicide.mp. or exp Suicide, Assisted/ or exp Suicide/ or exp Suicide, Attempted/	82,015
13	8 or 9 or 10 or 11 or 12	1,283,030
14	exp Cognitive Behavioral Therapy/ or CBT.mp.	30,605
15	cognitive processing therapy.mp.	288
16	exp Psychotherapy, Multiple/ or exp Psychotherapy, Group/ or exp Psychotherapy, Rational-Emotive/ or exp Psychotherapy, Brief/ or psychotherapy.mp. or exp Psychotherapy, Psychodynamic/ or exp Psychotherapy/ or exp “Imagery (Psychotherapy)”/ or exp Person-Centered Psychotherapy/	197,969
17	EMDR.mp. or exp Eye Movement Desensitization Reprocessing/	567
18	prolonged exposure.mp.	9683
19	psychological debriefing.mp.	123
20	exp Behavior Therapy/ or stress inoculation training.mp.	71,526
21	seeking safety.mp.	95
22	14 or 15 or 16 or 17 or 18 or 19 or 20 or 21	210,931
23	7 and 13 and 22	803
24	limit 23 to (English language and humans and “all adult (19 plus years)” and (adaptive clinical trial or clinical trial, all or clinical trial, phase i or clinical trial, phase ii or clinical trial, phase iii or clinical trial, phase iv or clinical trial or controlled clinical trial or randomized controlled trial))	31

**Table 4:** PsycINFO: September 28, 2019

Step	Search	Hits
1	exp First Responders/ or first responder.mp.	313
2	exp Law Enforcement/ or exp Police Personnel/ or police.mp.	43,130
3	exp Fire Fighters/ or exp Emergency Services/	10,978
4	prison.mp. or exp Prisons/	17,321
5	exp Corrections Officers/	458
6	exp Police Personnel/ or dispatch.mp.	9173
7	paramedic.mp. or exp Paramedics/	389
8	1 or 2 or 3 or 4 or 5 or 6 or 7	68,348
9	exp Occupational Stress/ or exp Posttraumatic Stress Disorder/ or exp Mental Health/ or operational stress.mp. or exp Stress Reactions/	120,758
10	exp Atypical Depression/ or exp Endogenous Depression/ or exp Intervention Resistant Depression/ or exp Major Depression/ or depression.mp.	315,162
11	mood disorder.mp. or exp Affective Disorders/	139,363
12	anxiety disorder.mp. or exp Anxiety Disorders/	59,104
13	substance abuse.mp. or exp Drug Abuse/	64,707
14	exp Drug Abuse/ or exp Personality Disorders/ or exp Drug Dependency/ or exp Alcoholism/ or exp Alcohol Abuse/ or exp Addiction/ or substance dependence.mp.	144,241
15	exp Attempted Suicide/ or exp Suicide Prevention Centers/ or exp Assisted Suicide/ or exp Suicide Prevention/ or exp Suicide/ or suicide.mp.	55,358
16	9 or 10 or 11 or 12 or 13 or 14 or 15	621,856
17	exp Cognitive Therapy/ or exp Cognitive Behavior Therapy/ or exp Group Psychotherapy/ or exp Computer-Assisted Therapy/ or cbt.mp.	63,467
18	exp Individual Psychotherapy/ or psychotherapy.mp. or exp Interpersonal Psychotherapy/ or exp Psychotherapy Training/ or exp Supportive Psychotherapy/ or exp Psychodynamic Psychotherapy/ or exp Psychotherapy/	239,022
19	exp Cognitive Processing Therapy/ or cognitive processing therapy.mp.	425
20	EMDR.mp. or exp Eye Movement Desensitization Therapy/	1761
21	exp Exposure Therapy/ or exp Prolonged Exposure Therapy/ or prolonged exposure.mp.	6076
22	exp “Debriefing (Psychological)”/ or debriefing.mp.	2641
23	exp Stress Management/ or exp Behavior Modification/ or exp Virtual Reality/ or stress inoculation training.mp. or exp Relaxation/	58179
24	seeking safety.mp.	177
25	17 or 18 or 19 or 20 or 21 or 22 or 23 or 24	321,375
26	8 and 16 and 25	970
27	limit 26 to (human and English language and yr= “2008–Current”)	429

**Table 5:** EMBASE: September 28, 2019

Step	Search	Hits
1	exp rescue personnel/ or exp emergency health service/ or first responder.mp.	104,258
2	exp emergency police dispatcher/ or exp police/	12,482
3	firefighter.mp. or exp firefighter/	3054
4	exp prison/ or correctional officer.mp.	16,543
5	paramedic.mp. or exp paramedical personnel/ or exp ambulance/	502,453
6	corrections.mp.	23,473
7	1 or 2 or 3 or 4 or 5 or 6	638,692
8	PTSD.mp. or exp posttraumatic stress disorder/	59,842
9	operational stress injury.mp.	9
10	mood disorder.mp. or exp mood disorder/	506,502
11	exp depression/ or depression.mp. or exp major depression/	708,471
12	anxiety disorder.mp. or exp anxiety disorder/	237,091
13	substance abuse.mp. or exp substance abuse/	67,576
14	exp addiction/ or addiction.mp.	325,666
15	drug dependence.mp. or exp drug dependence/	248,881
16	exp suicide/ or suicide.mp. or exp suicide attempt/	108,879
17	8 or 9 or 10 or 11 or 12 or 13 or 14 or 15 or 16	1,253,742
18	exp cognitive therapy/ or exp behaviour therapy/ or cbt.mp. or exp cognitive behavioural therapy/	91,611
19	exp psychotherapy/ or psychotherapy.mp. or exp psychodynamic psychotherapy/ or exp short term psychotherapy/	271,424
20	cognitive processing therapy.mp.	360
21	exp “eye movement desensitization and reprocessing”/ or exp eye movement/ or EMDR.mp.	57,185
22	prolonged exposure.mp. or exp long term exposure/	37,507
23	exp inoculation/ or stress inoculation training.mp.	49,168
4	exp crisis intervention/ or debriefing.mp.	11,408
25	seeking safety.mp.	124
26	18 or 19 or 20 or 21 or 22 or 23 or 24 or 25	426,787
27	7 and 17 and 26	1583
28	Limit 27 to (human and English language and yr=“2008–Current”)	1003

## Appendix 2

Table 6

**Table 6:** Risk of bias assessment using the Cochrane Risk of Bias Tool

Study	Randomization	Allocation	Blinding	Attrition	Selective reporting	Other bias
Alghamdi et al. 2015 [76]	Low risk	Unclear risk	High risk	Low risk	Low risk	Low risk
Bryant et al. 2019 [71]	Low risk	Low risk	Low risk	Low risk	Low risk	Low risk
Chongruksa et al. 2012 [73]	Unclear risk	Unclear risk	High risk	Unclear risk	Low risk	Unclear risk
Gersons et al. 2013 [72]	Low risk	Low risk	Low risk	Low risk	Low risk	Low risk
Jarero et al. 2013 [75]	Low risk	Unclear risk	High risk	Low risk	Low risk	High risk
Miller et al. 2019 [77]	Low risk	Unclear risk	High risk	Low risk	Low risk	Low risk
Mithoefer et al. 2018 [70]	Low risk	Low risk	Low risk	Low risk	Low risk	Low risk
Tuckey et al. 2014 [74]	Low risk	Low risk	High risk	Low risk	Low risk	High risk

## Appendix 3

Table 7

**Table 7:** Summary of results

Outcome	Effect size	95% CI	Studies
PTSD severity, endpoint	*d* = − 1.23	− 1.81, − 0.675	7
PTSD severity, follow-up	*d* = − 1.29	− 2.31, − 0.27	6
PTSD remission, endpoint	RR = 1.81	1.18, 2.79	2
PTSD remission, follow-up	RR = 2.15	1.13, 4.11	2
Anxiety severity, endpoint	*d* = − 0.76	− 1.28, − 0.24	3
Anxiety severity, follow-up	*d* = − 0.82	− 1.20, − 0.44	3
Depression severity, endpoint	*d* = − 1.10	− 1.62, − 0.58	5
Depression severity, follow-up	*d* = − 0.46	− 0.77, − 0.14	5
Acceptability, endpoint	RR = 1.00	0.96, 1.05	8
Acceptability, follow-up	RR = 1.00	0.95, 1.05	8

*Abbreviations*: *PTSD* post-traumatic stress disorder, *d* Cohen’s *d* standardized mean difference, *RR* rate ratio

## Appendix 4

Figure 5

## Appendix 5

Figure 6
